# Cell Lineage Determination in State Space: A Systems View Brings Flexibility to Dogmatic Canonical Rules

**DOI:** 10.1371/journal.pbio.1000380

**Published:** 2010-05-25

**Authors:** Sui Huang

**Affiliations:** Institute for Biocomplexity and Informatics, University of Calgary, Calgary, Canada

*A model of cell-type diversification that allows for stochastic sampling of available developmental states reconciles real-world observations with the seemingly incongruent rules of deterministic cell fate decision making.*

The quest for the origin of diversity in biology is as old as the discipline itself. Much as evolutionary biologists sought to fathom the diversification of life forms into discrete, self-maintaining species, developmental biologists are now trying to understand the diversification of embryonic cells into discrete, self-maintaining cell types of the multicellular body. This challenge revolves in large part around the following questions: how does the fertilized egg—a single cell—generate all cell lineages, including those that build the extra-embryonic tissues; and how can apparently uniform embryonic stem (ES) cells (i.e., cells derived from the inner cell mass of the early embryo) give rise to all the cell types of the adult [1]? The answers depend on understanding how the *totipotent* fertilized egg and *pluripotent* embryonic stem cells lose their potentials as their cellular progenies differentiate into distinct types during development.

Ironically, history repeats itself. Just as evolutionary theory had to overcome the inexorable idea of immutability of species and establish the role of “chance” in evolution, stem cell biology has to overcome the dogma that the fate of cells, once achieved, is irreversible [2]. Even more engrained is the notion that cell fate is determined by tightly controlling regulatory pathways that coordinate, like a well-designed clock, the intertwined molecular mechanisms that underlie specific cell phenotypes. This so-called deterministic view [3], which still dominates modern molecular biology, not only doubts cell phenotypic plasticity but also eschews stochastic (i.e., random) processes. Deterministic thinking has led to an edifice of qualitative ad hoc concepts and black-and-white canonical rules describing, for example, the lawful developmental relationship between particular cell types (which cell is derived from which one) and circumscribed stages of commitment (blank state, primed, determination, commitment, etc.).

Mounting evidence has revealed the limitations of this deterministic view. First, as systems biology has championed the notion of “gene expression noise” [4],[5], the idea of chance events in cell fate decisions, first boldly proposed by Kupiec in the 1980s, has become acceptable [6],[7]. “Gene expression noise”—the stochastic temporal variations of gene expression levels—is thought to arise when the small number of molecules involved in a biomolecular chemical reaction in the cell, such as transcription, prevents natural fluctuations from “averaging out.” It hence brings the thermal fluctuations inherent in chemical reactions into the realm of biology which makes stochasticity an inevitable aspect of the physics of cells. Second, the accumulating evidence of transcriptionally induced transdifferentiation between lineages—pioneered by Weintraub [8] and Graf [9] and culminating in the recently achieved, easily reproduced reprogramming of adult cells into “induced pluripotent stem” (iPS) cells by overexpression of pluripotency transcription factors [10]—has refuted the dogma of cell fate irreversibility

## Choosing between Primitive Endoderm and Epiblast: Not Black and White

The article by Brickman and coworkers (Canham et al., this issue of *PLoS Biology* [11]) is the latest in a recent series of reports (reviewed in [12] and [13]; [14]–[18]) that have begun to challenge the view that embryonic cells undergo a tightly controlled, predestined one-way journey from totipotency to pluripotency towards specific lineages. Brickman's work undercuts this paradigm in two ways. First, it was long thought that inner cell mass (ICM)–derived ES cells cannot contribute to the primitive endoderm (PE), which forms extra-embryonic endodermal tissues, such as the yolk sac—i.e., that ES cells are pluri- but not totipotent [1]. Second, the deterministic view tacitly assumes that cells commit to a lineage because specific dedicated environmental signals (position in the embryo, cytokines, cell–cell interactions, etc.) instruct them to do so. Rather than following strict orders, however, ES cells may be capable of more flexible behaviour [19],[20], such that randomly vacillating ES cells, which in this case are expected to become epiblast (precursor of the embryo proper), may transiently entertain, in a barely detectable fashion, the possibility of choosing a PE fate instead.

Immunostaining or fluorescent proteins that report the expression of lineage-specific markers have revealed the extensive heterogeneity of cells within the early embryo [13], which investigators have interpreted as a manifestation of gene expression noise, hence indicating stochasticity [14]. (Reality may be more complex, as discussed below.) Canham et al., using a particularly sensitive reporter, detect a subpopulation within ES cells that express Hex, an early marker of the PE lineage. Importantly, through sorting and repopulation experiments they confirm that the heterogeneity of ES cells is not static but represents a snapshot of a dynamic equilibrium [12],[21] in which ES cells oscillate between different “primed states,” poised to become PE at one point and epiblast at another.

The dominance of the canonical rules in development may recede with the advent of systems biology which aspires to describe biological phenomena in terms of fundamental principles of physics and mathematics. This in turn requires that observations be explained as a necessary consequence of these principles instead of using ad hoc rules.

## Fundamental Principles of Complexity in Dynamical Systems: Toward a Formal Theory of Cell Fate Determination

**Figure 1:**
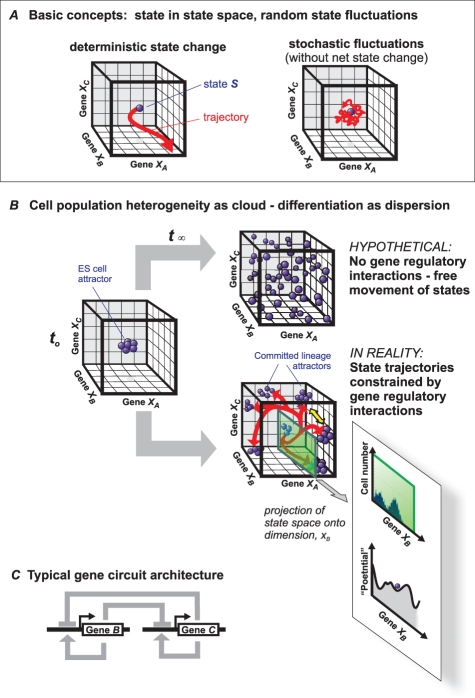
Fundamental principles of high-dimensional dynamical systems that may explain the coordinated change of gene expression during cell fate commitment and phenotype change and integrates chance and necessity. (A) Basic concepts. The “cube” represents a three-dimensional state space (describing a three-gene system (genes *A*, *B*, and *C*) with their expression levels (*x_A_*, *x_B_*, and *x_C_*) as axes. A state *S* is a point in state space (blue ball). When gene expression pattern changes, the state moves along a trajectory. If gene *B*, which suppresses gene *C*, increases its expression *x_B_*, then the point *S* will move in the direction of the axis of increasing *x_B_* and at the same time, by necessity, of decreasing *x_C_*. (B) Application of state space and cell state concepts to a population of cells represented by a “cloud” of states. The interaction between the genes (state space dimensions) prevents the hypothetical even dispersion into the entire state space, instead allowing cells to occupy only predestined regions (cell type attractors) by following the trajectories (red). The mutual inhibition of *x_B_* and *x_C_*, for instance, pushes cells away towards an [*x_B_*≫*x_C_*] and an [*x_B_*≪*x_C_*] attractor. Yellow double arrow indicates the trajectory separation. For details see text. The insets at the bottom represent a histogram as typically observed in flow cytometry, which represents a projection of the state space for *X*_B_ and the quasi-potential landscape (schematically) along *X*_B_. Note that because this is a non-integrable, non-conservative system, the elevation of the landscape does not represent true potential energy. (C) Example of a typical gene regulatory circuit of two mutually inhibiting and self-activating genes *B* and *C* (for instance Gata6 and Nanog) that establishes a metastable bipotent state *x_B_*≈*x_C_* that can differentiate into either one of the two committed lineage attractors, [*x_B_*≫*x_C_*] and [*x_B_*≪*x_C_*].

Certain inescapable fundamental principles underlie the genetic regulation of cell development (allowing here for a simplifying abstraction). A gene expression pattern across the genome constitutes a *cell state “S”*, and any cell phenotype change away from this state is in principle the coordinated change of expression of the genes that define the state. A cell state *S* = [*x_1_*, *x_2_*, …*x_i_*, …*x_N_*] is jointly defined by the values *x_1_*, *x_2_*, …*x_i_*, …*x_N_* that represent the expression levels of the genes *1*, gene *2*, gene *i*, etc. in a genome of *N* genes. The concept of *state space* lies at the core of the theory of dynamical systems. (See Figure 1A for an example of a three-gene state space.) Each state is a point in this state space where the gene expression values *x_i_* defining a particular state *S* are interpreted as coordinates to define the position of that state. Distinct cell types would occupy different regions of the state space. A change in expression pattern corresponds with the restricted movement of *S* in the state space along a *trajectory*. This movement is restricted because of gene interactions: for instance, if a gene *A* inhibits gene *B*, then as *A* increases its expression, *B* necessarily *has* to decrease.

Let us now assume that an embryonic stem cell is represented by a state *S_ES_* at the center of a given state space. Due to gene expression noise, the values of all *x_i_* will fluctuate randomly, thereby causing *S* to describe an erratic trajectory referred to as a “random walk” (Figure 1A). As cells divide, new points are generated, each of which designates a new, independent cell and its state. This leads to a *cloud of points* that gradually expands due to the random fluctuations in each cell (Figure 1B). They will eventually fill the entire state space, much as gas molecules concentrated in the center of a container, when released, would inevitably fill the container due to the random thermal fluctuations of the molecules. Thus, in this hypothetical scenario we have now a crude formal description of the inevitable (but uncontrolled) diversification of gene expression patterns within a population of cells that is driven by random events.

But in reality, cell type diversification creates only a small subset of states among all the possible states because, unlike a gas in physical space, a cell state *S* does not move entirely randomly; the individual genes do not alter their expression value *x_i_* independently because of predetermined regulatory interactions. Hence, the change of gene expression patterns as a whole (i.e., the trajectory of *S*) is highly constrained. This means that expansion of the cloud of states into the state space is not uniform but channelled, namely towards state space regions that represent expression patterns in which all regulatory interactions are “satisfied.” Called *attractor states*, these states are stable and attract the points in their state space neighbourhood away from states that represent unstable gene expression patterns because of conflicting gene expression configurations (e.g., gene *A* high *and* gene *B* high although *A* inhibits *B*). In 1969, Stuart Kauffman first proposed that cell types are attractors of the network [22]. Only recently has experimental evidence of this prescient idea been obtained (reviewed in [23]). Cells are most likely to be found at the center of an attractor, and less likely at the periphery. This probabilistic view allows one to (crudely) compute a (quasi) potential landscape where stable (hence, probable) attractor states correspond to valleys (Figure 1B inset bottom) [24],[25].

## Heterogeneity: Clouds in State Space and Their Restriction by Regulatory Circuits

Because of gene expression noise, any cell population of the same cell type (e.g., ES cells) is not represented by one point *S* in state space but covers a cloud of points – or a set of similar expression profiles. In one state space dimension, this can be represented by the familiar histogram (Fig. 1B, inset). The dispersion of a uniform cell population into a cloud is a static snapshot of a group of cells asynchronously fluctuating in state space, held together because of an attractor akin to a swarm of flies flying around a light. Thus, the cloud of states represents a dynamical equilibrium between dispersing forces and attracting forces. The latter ensures an on-average characteristic gene expression pattern, such as *S_ES_* for ES cells.

This picture provides a formal model derived from the mathematical treatment of gene regulatory interactions. Although we massively simplified the theory here, it captures the inevitable “entropy-driven” dispersion that inevitably promotes diversification of phenotypes into discrete clusters.

Applying this general picture to fate determination in ES cells, the factors that control major decisions between opposing cell lineages inhibit each other, thus generating complementary expression patterns and pushing trajectories away to opposing corners of the state space (Figure 1B). In the fate decision of 3.5-day-old embryos, this is accomplished by the transcription factors Nanog and GATA6, which inhibit each other and promote either the epiblast or the PE fate, respectively [26],[27]. Such mutually repressing transcription factors (Genes *B* and *C* in Figure 1B) typically govern a behaviour such that the asymmetric expression patterns, either [*x_B_*≫*x_C_*] or [*x_B_*≪*x_C_*], represent stable, mutually exclusive fates [27]. However, in such “bistable” systems [28], the balanced central state [*x_B_*≈*x_C_*] would not be stable unless there is also an autostimulatory feedback loop that controls these two opposing transcription factors [29]. Evidence for such autoregulatory feedback loops in fact is almost ubiquitously found to be a property of transcription factors involved in fate decisions [30]–[34]. Mathematical modeling shows that they render the unstable, balanced state partially stable (“metastable”) [24],[29],[35] (Figure 1C). This “attracting” component explains how the pluripotent state (the “ground state character” [36]) can be maintained in the absence of differentiating signals. Differentiation signals then have been proposed to *destabilize* this attractor state [24],[29], forcing the cloud to disperse towards the two stable states. In this model, instructive signals bias the cloud towards either one of the two available stable states. This concept naturally integrates stochastic and deterministic fate decisions.

## A New More Flexible, Less Dogmatic Picture

The picture of a cloud in state space provides a new conceptual tool for thinking about fate determination of pluripotent cells. Together with increasingly sensitive single-cell techniques, we begin to appreciate that “outlier cells” in apparently uniform cell populations are not just statistical blips but pioneers in state space exploration [37]. Among the ES cells described by Canham et al., those with slightly higher expression of Hex (signaling a tad higher amount of Gata6 than Nanog) that are not yet committed would be poised to commit to the PE lineage, primed to receive the appropriate instructive signals that would channel them toward that particular stable state space region. In the absence of signals, however, they would most likely swing back to the ground state at the center of the cloud, due to the attractor property. This could be one manifestation of a more general principle according to which each region in the state space cloud of the embryonic cells' attractor may contain cells differentially poised for different fates which dynamically interconvert [15]. This set of indeterminate states within a centrally located meta-stable state space region that has channel connections to various peripheral regions may be the very essence of pluripotency.

One interesting question emanating from the formal concept of a cloud is whether its essential dispersion in state space is simply a function of “gene expression noise.” Recent work suggests that reality is more complex: the fluctuations are rather slow and richly structured [37], which is not surprising given the complexity of the gene regulatory network across the genome [21]. First, the ES cell attractor is in a high-dimensional space and of unknown shape, and thus, the ground state may consist of a set of distinguishable sub-states; its characterization based on particular markers, such as Nanog or Hex, however, is akin to examining a complex structure along one cross section or by projection into one plane (Figure 1B, bottom) which abnegates substantial information. Also, specific network dynamics may be dedicated to either promoting exploration of marginal regions [20] or to limiting its dispersion, akin to “noise suppression,” which would stabilize the ground state and hence maintain pluripotency [38]. Thus, the artificial conditions in cell culture that define ES cells may fail to recapitulate the physiological cascade of events in the embryo environment that would naturally disperse and destabilize the cloud of pluripotent states, forcing cells to follow entropy and, in their journey that is constrained by the gene regulatory network interactions, populate the various peripheral regions of the state space.

In this sense, the metaphor used by Canham et al. that ES cells are “trapped” in equilibrium between various short-lived explorative states, is quite adequate. However, it can be linked to a deeper conceptualization in which expression pattern fluctuations are constrained by attractors in state space, leading to the formation of compact clouds of dynamical states that fail to “dissolve” for lack of the appropriate destabilizing conditions. This general picture, however, is not a metaphor, but grounded in first principles of physical dynamical systems. It unites deterministic and stochastic mechanisms. While it still needs to be filled with specific molecular details, this formal conceptual framework will hopefully help to accommodate the continuing discovery of unexpected lineage conversions that new sensitive technologies reveal—conversions that we can no longer ignore because they do not confirm to traditional black-and-white canonical rules of developmental biology.

## Abbreviations

ES: embryonic stem
iPS: induced pluripotent stem
ICM: inner cell mass
PE: primitive endoderm
